# The In Vitro Transgenic Rodent Assay in Primary MutaMouse Hepatocytes Compared to the Mammalian Cell Gene Mutation Assay Using the HPRT Gene

**DOI:** 10.1002/em.70040

**Published:** 2025-10-29

**Authors:** Alina Göpfert, Kylee Kendra Marie Ronnenberg, Claudia Ruelker, Silke Spang, Naveed Honarvar, Robert Landsiedel

**Affiliations:** ^1^ BASF SE Experimental Toxicology and Ecology Ludwigshafen am Rhein Germany

**Keywords:** benchmark concentration modeling, gene mutation, GentleMACS, liver perfusion, mutagen, validation

## Abstract

Gene mutations can be detected in mammalian cells in vitro using indicator genes such as the hypoxanthine‐guanine‐phosphoribosyltransferase (HPRT) gene. These assays have been adopted as OECD test guidelines (TG, e.g., OECD TG no. 476) and are used for regulatory purposes. The in vitro transgenic rodent assay (TGRA) in primary MutaMouse hepatocytes is a novel approach for the detection and quantification of gene mutations. Its methodology follows the same principles as the in vivo TGRA, an in vivo gene mutation assay with regulatory adoption (OECD TG no. 488). Although the potential of the in vitro TGRA to identify mutagens has been reported, its performance compared to an established in vitro gene mutation assay has not been reported. This study compared the in vitro TGRA with the HPRT assay using 10 known in vivo mutagens. The in vitro TGRA correctly identified all 10 mutagens, whereas the HPRT assay identified only nine. Benchmark concentration (BMC) modeling for the nine substances detected by both assays revealed overlapping confidence intervals for six compounds, indicating comparable sensitivity. For three mutagens, the HPRT assay yielded lower BMC intervals. Additionally, eight substances known to be non‐mutagenic in vivo tested negative in the in vitro TGRA. While increased cytotoxicity did not induce increased mutant frequencies, it reduced DNA yield, thereby impairing mutagenicity assessment. The results of this study contribute to the understanding of the sensitivity and robustness of the in vitro TGRA and provide essential information for the validation of the assay.

## 1 Introduction

To align with the 3R principle (replacement, reduction, and refinement of animal studies) proposed by Russell and Burch (Russell and Burch 1959), the scientific community is working to reduce the number of animals used in toxicology testing (Kirkland et al. 2007; OECD 2017). For the endpoint of mutagenicity, regulatory toxicology testing strategies across different jurisdictions recommend the use of two or three in vitro tests, followed by as few in vivo tests as possible (Cimino 2006; Kirkland et al. 2007; OECD 2017). For the regulatory assessment of gene mutation induction in vitro, internationally accepted assays are available with test guidelines (TG) from the Organization for Economic Cooperation and Development (OECD). These assays are performed either in bacteria (bacterial reverse mutation test, TG 471 (OECD 2020)) or in mammalian cell lines (in vitro mammalian cell gene mutation test using the Hprt/xprt genes or the Thymidine Kinase gene, TG 476 (OECD 2016a) and TG 490 (OECD 2016b)) (OECD 2017). While these in vitro gene mutation assays have been validated and used in hazard assessment for a long time, a positive result from a single in vitro assay is often not reflected in in vivo follow‐up assays (Fowler et al. 2024; Kirkland et al. 2007).

The in vivo transgenic rodent assay (*vivo*TGRA), described by OECD TG No. 488 (OECD 2022), is a widely used animal test to follow up positive results obtained from in vitro gene mutation assays. Previous studies (Chen et al. 2010; Cox et al. 2019a, 2019b; Luijten et al. 2016; White et al. 2003; Zwart et al. 2012) have introduced an in vitro analog of the *vivo*TGRA. The experimental procedure of the in vitro TGRA (*vitro*TGRA) is the same as that of its in vivo counterpart, but cells isolated from transgenic rodents are incubated with the test substance in vitro rather than administering the test substance to the transgenic animals. Recent studies have shown that the *vitro*TGRA in primary hepatocytes could be a suitable mammalian cell gene mutation assay to complement the current test battery of in vitro assays for mutagenicity assessment (Chen et al. 2010; Cox et al. 2019b; Göpfert et al. 2025; Zwart et al. 2012). Unlike other currently available in vitro mammalian cell gene mutation assays, the *vitro*TGRA detects gene mutations in the same reporter gene and with the same readout as the *vivo*TGRA. Furthermore, by using primary hepatocytes from transgenic animals, the test system possesses the xenobiotic‐metabolizing capacity of a liver cell (Cox et al. 2019a). Thus, the *vitro*TGRA might better predict the in vivo outcome compared to the other established in vitro mammalian cell gene mutation assays, which rely on exogenous liver homogenate.

One aim of this study was to compare the performance of the *vitro*TGRA with the well‐established in vitro mammalian cell gene mutation test using the Hprt gene (HPRT assay). This provides information on the sensitivity of the *vitroTGRA*, which is an essential component for establishing its context of use. The second aim of the study was to address the specificity of this novel assay. The upper concentration tested in an in vitro assay, is an experimental condition influencing this. The upper test concentration can be defined by toxicokinetic considerations, the solubility of the test substance, or its cytotoxicity (Landsiedel et al. 2024). The test guidelines for in vitro mammalian cell gene mutation assays using the Hprt, Xprt, and thymidine kinase genes recommend not exceeding a concentration of the test substance that causes cytotoxicity (measured as a reduction in relative survival (RS) or relative total growth (RTG)) to approximately 10%–20% of the concurrent solvent control (OECD 2016a, 2016b). Exceeding this cytotoxicity threshold is considered excessive and may complicate the interpretation of the test results (OECD 2016a, 2016b). Cox et al. (2019b) recommended adopting this threshold for the *vitro*TGRA. However, it is not known whether this threshold is appropriate for the *vitro*TGRA. To determine appropriate concentration selection for cytotoxic test substances in the *vitro*TGRA, non‐DNA‐reactive substances were selected and tested at high, cytotoxic concentrations.

## 2 Materials and Methods

### 2.1 Materials

#### 2.1.1 Chemicals and Reagents

Cell culture media (Dulbecco's Modified Eagle's Medium—High Glucose (DMEM), Williams Medium E, DMEM Ham's F12), Fetal Calf Serum (FCS), HEPES buffer, Hank's Balanced Salt Solution (HBSS) and 10,000 U/mL Penicillin/10 mg/mL Streptomycin (Pen/Strep) were purchased from PAN Biotech, Aidenbach, Germany. Additives and cofactors were obtained from Merck KGaA, Germany (L‐proline, L‐glutamine, sodium pyruvate, ethylene glycol tetraacetic acid (EGTA), ethylenediamine tetraacetic acid (EDTA), human insulin (h‐insulin), dexamethasone, sodium hydroxide, Na_2_HPO_4_, NaH_2_PO_4_, calcium chloride, potassium chloride, and magnesium chloride); Thermo Fisher Scientific, Bremen, Germany (Gibco mouse epidermal growth factor (mEGF)); or Roche, Germany (Glucose‐6‐phosphate Na_2_, Glucose‐6‐phosphate dehydrogenase, NADP Na_2_, NADH Na_2_). The S9 mix derived from phenobarbital/ß‐naphthoflavone‐induced rat livers was purchased from ICCR Roßdorf GmbH, Roßdorf, Germany, and tested for its activity to benzo[*a*]pyrene and acetamidofluorene. Collagen I‐coated petri dishes were purchased from Corning Inc. (New York, USA), and collagenase HA and protease BP were obtained from VitaCyte LLC (distributed by PELObiotech GmbH, Planegg, Germany). The liver Perfusion Kit, mouse and rat and the MACS Tissue Storage Solution were obtained from Miltenyi Biotec B.V. & Co. KG, Bergisch Gladbach, Germany. Reagent‐grade dimethyl sulfoxide (DMSO) was purchased from AppliChem, Darmstadt, Germany. Reagents used for cell lysis, DNA isolation, flow cytometry, and phage packaging were purchased from Merck KGaA, Darmstadt, Germany (proteinase K, IGEPAL‐ CA‐630, tris(hydroxymethyl)aminomethane (TRIS), trisodium citrate, sodium dodecyl sulfate (SDS), buffer‐saturated phenol, chloroform: isoamyl alcohol (24:1), 2% (w/v) gelatin solution, 100 mg/mL ampicillin, kanamycin sulfate, *N*,*N*‐dimethylformamide (DMF), yeast extract, tryptone, maltose monohydrate); Honeywell Fluka, Seelze, Germany (sucrose, sodium chloride (NaCl), ethanol, magnesium sulfate heptahydrate (MgSO_4_*7 H_2_O)); Invitrogen, distributed by Thermo Fisher Scientific, Bremen, Germany (20 mg/mL PureLink Ribonuclease A, SYTOX Green, Cellsorting Set up Beads for Blue Lasers); Bernd Kraft, Duisburg, Germany (citric acid monohydrate); Becton Dickinson, Heidelberg, Germany (Bacto Agar); and VWR, Darmstadt, Germany (phenyl‐β‐D‐galactopyranoside (P‐Gal)). Sheath Fluid and FACS Clean were obtained from BD Biosciences, Heidelberg, Germany. The Transpack packaging extract for Lambda Transgenic Shuttle Vector Recovery was purchased from Agilent Technologies Inc., Waldbronn, Germany.

#### 2.1.2 Test Substances

Based on the recommendations for the “assessment of the performance of new or improved genotoxicity tests” by Kirkland et al. (2016), eight non‐DNA‐reactive substances were selected to address potential confounding cytotoxic effects during the mutagenicity assessment in the *vitroTGRA* using primary MutaMouse hepatocytes. The eight substances (benzyl alcohol, diclofenac sodium (hereafter referred to as diclofenac), erythromycin, ethionamide, eugenol, methyl carbamate, sulfisoxazole, and urea) gave negative results in in vivo genotoxicity assays. Benzyl alcohol, ethionamide, eugenol, sulfisoxazole, and urea have been reported to induce mutations in other in vitro mammalian cell mutagenicity assays (Kirkland et al. 2016).

Ten substances known to give positive results in in vivo genotoxicity assays were selected. These were 2‐acetylaminofluorene (2‐AAF), 4‐nitroquinoline‐1‐oxide (4‐NQO), benzo[a]pyrene (B[a]P), cyclophosphamide (CPA), 7,12‐dimethylbenz[a]anthracene (DMBA), ethyl methanesulfonate (EMS), N‐ethyl‐N‐nitrosourea (ENU), 3‐methylcholanthrene (MCA), mitomycin C (MMC) and 2‐amino‐1‐methyl‐6‐phenylimidazo[4,5‐b]pyridine (PhIP). Of these, 2‐AAF, B[*a*]P, CPA, DMBA, MCA, and PhIP required external metabolic activation (by S9‐mix) to exhibit mutagenic effects in the HPRT‐assay.

Stock solutions of CPA, MMC, methyl carbamate and urea were prepared in culture medium; stock solutions of benzyl alcohol were prepared in water (10% final in culture medium); and stock solutions of 2‐AAF, 4‐NQO, B[*a*]P, diclofenac, DMBA, EMS, ENU, erythromycin, ethionamide, eugenol, MCA, sulfisoxazole, and PhIP were prepared in DMSO (1% final in culture medium). For both in vitro gene mutation assays, test concentrations for the main experiments were based on preliminary experiments (data not shown). The concentration selection was based on the criteria of the OECD TG for the HPRT assay (No. 476). The highest test concentration was set at 10 mM or 2 mg/mL, whichever value was lower (OECD 2016a). For poorly soluble substances, the highest tested concentration was the lowest precipitating concentration (OECD 2016a). For cytotoxic mutagens tested in the *vivo*TGRA and HPRT assay, the highest concentration evaluated for mutagenicity was required to induce 80%–90% cytotoxicity (OECD 2016a). For non‐DNA‐reactive substances evaluated exclusively in the *vivo*TGRA, cytotoxicity was not considered when selecting test concentrations. CAS numbers, suppliers of the test substances, solvents used to prepare stock solutions, and the requirement for exogenous metabolic activation (use of S9 mix) in the HPRT assay are listed in Table 1. All test substances were ordered with a purity of > 98%.

**TABLE 1:** Suppliers of the test substances tested in the *vitro*TGRA and the HPRT assay with additional information on the solvent used and the requirement for S9 mix in the HPRT assay.

Test substance	CAS‐number	Supplier	Solvent	In vivo genotoxin	S9
Benzyl alcohol	100‐51‐6	Sigma‐Aldrich	Water	—	^a^
Diclofenac	15307‐79‐6	Sigma‐Aldrich	DMSO	—	
Erythromycin	114‐07‐8	Sigma‐Aldrich	DMSO	—	
Ethionamide	536‐33‐4	Sigma‐Aldrich	DMSO	—	
Eugenol	97‐53‐0	Sigma‐Aldrich	DMSO	—	
Methyl carbamate	598‐55‐0	Sigma‐Aldrich	Culture medium	—	
Sulfisoxazole	127‐69‐5	Sigma‐Aldrich	DMSO	—	
Urea	57‐13‐6	Sigma Aldrich	Culture medium	—	
2‐AAF	53‐96‐3	Sigma‐Aldrich	DMSO	+	+
4‐NQO	56‐57‐5	Sigma‐Aldrich	DMSO	+	—
B[*a*]P	50‐32‐8	Sigma‐Aldrich	DMSO	+	+
CPA	50‐18‐0	Baxter	Culture medium	+	+
DMBA	57‐97‐6	Sigma‐Aldrich	DMSO	+	+
EMS	62‐50‐0	Sigma‐Aldrich	DMSO	+	—
ENU	759‐73‐9	Sigma‐Aldrich	DMSO	+	—
MCA	56‐49‐5	Sigma‐Aldrich	DMSO	+	+
MMC	50‐07‐7	Thermo Scientific	Culture medium	+	—
PhIP	105650‐23‐5	Cayman Chemical	DMSO	+	+

^a^ These substances were not tested in the HPRT assay in this study.

### 2.2 Methods

#### 2.2.1 In Vitro Transgenic Rodent Gene Mutation Assay ( *vitro*TGRA) Using Primary MutaMouse Hepatocytes

##### 2.2.1.1 Test System

For isolation of primary hepatocytes (PHs), female mice of the transgenic MutaMouse strain 40.6 were purchased from Laboratory Corporation of America Holdings (LabCorp, United Kingdom). The mice were housed locally in accordance with the standards set by the Association for Assessment and Accreditation of Laboratory Animal Care (AAALAC). The mice were kept in type II polycarbonate cages in a group housing environment within a fully air‐conditioned, specific‐pathogen free room. They were maintained on a 12‐h light–dark cycle, with the temperature kept between 20°C–24°C. Relative humidity was constantly between 45%–65%. The mice had ad libitum access to standardized pelleted feed (Granovit AG, Kaiseraugst, Switzerland) and drinking water. Mice were held for at least 5 days for acclimatization prior to hepatocyte isolation.

##### 2.2.1.2 Isolation of Primary MutaMouse Hepatocytes

Primary hepatocytes (PHs) were isolated from 7‐ to 24‐week‐old female MutaMouse specimens either by a two‐step retrograde liver perfusion or by using the gentleMACS Perfusion Technology from Miltenyi Biotech. The two‐step retrograde liver perfusion was performed as described previously (Göpfert et al. 2025). Briefly, mice were anesthetized with Narcoren (i.p., 213 mg pentobarbital sodium/kg body weight) and exsanguinated via whole‐body perfusion with blanch solution (HBSS supplemented with 10 mM HEPES, 1 mM EGTA, and 100 U/mL penicillin–streptomycin). To perfuse the liver, the vena cava inferior was accessed via the right atrium. The two‐step retrograde liver perfusion was initiated with 2–3 min of blanching, followed by a 15–20 min perfusion using collagenase HA (2000 U/mL) and protease BP (250 U/mL) containing DMEM medium. The perfused liver was then excised and rinsed in attachment medium (DMEM supplemented with 20 U/L human insulin, 4 × 10^−6^ mg/mL dexamethasone, 10% FBS, and 100 U/mL penicillin–streptomycin). The cells were released into the attachment medium by carefully disaggregating the liver capsule.

For the isolation of viable PHs using the gentleMACS Perfusion Technology from Miltenyi Biotec, the manufacturer's protocol was followed as described by Poggel et al. (2022). Mice were anesthetized by respiratory anesthesia of isoflurane and sacrificed by cervical dislocation. The liver was carefully excised from the abdominal cavity, placed into a petri dish filled with MACS Tissue Storage Solution, and the gall bladder was removed. To increase cell yield, the left and right lateral liver lobes were processed in parallel for each animal. Both liver lobes were dissected using a scalpel, and each lobe was individually secured between the grid and the clamp of a gentleMACS Perfuser Tube. The assembly of grid, liver lobe and clamp was attached to the lid and then screwed onto the bases of the gentleMACS Perfuser Tubes installed on the gentleMACS Octo Dissociator. Once the 37C_m_LIPK_1 program of the gentleMACS Octo Dissociator was initiated, an automated perfusion consisting of different steps—including washing, equilibration and enzymatic perfusion—was performed in sequence. Between steps, used buffers were replaced with fresh buffers, and for the enzymatic perfusion, an enzyme‐containing digestion mix was added to the tissue. Buffers and enzymes were provided in the Liver Perfusion Kit mouse and rat by Miltenyi Biotec. Following enzymatic perfusion, the enzyme‐containing digestion mix was transferred into a gentleMACS C tube along with the perfused tissue. To release the viable hepatocytes, the gentleMACS C tubes were installed on the gentleMACS OctoDissociator and automated tissue dissociation was initiated using the LIPK_HR_1 program.

Independent of the isolation method used, tissue debris was subsequently removed from the cells by filtering the suspension through a 100 μm cell strainer, followed by a 5 min centrifugation at 30 *g* and 4°C. The supernatant was aspirated, and the cell pellets were resuspended in attachment medium. The yield of viable primary hepatocytes was determined using the trypan blue exclusion method.

##### 2.2.1.3 Mutagenicity Assessment

The assay was performed as previously described by Göpfert et al. (Göpfert et al. 2025). Briefly, isolated primary MutaMouse hepatocytes were plated at a density of 800,000 cells per 100 mm collagen‐I‐coated petri dish in attachment medium. Two hours post plating, the attachment medium was replaced with serum‐free medium (SFM, 10 mM HEPES, 2 mM L‐glutamine, 10 mM pyruvate, 0.35 mM L‐proline, 20 U/L human insulin, 4 × 10^6^ mg/mL dexamethasone, 0.01 μg/mL mEGF, and 100 U/mL penicillin–streptomycin in Williams medium E), and the cells were cultured overnight. Unless otherwise stated, incubation conditions were as follows: 37°C in an atmosphere of 5.0% (v/v) CO_2_ and a relative humidity of ≥ 90%. The next morning, cells were treated with various concentrations of test substance and the respective vehicle for 6 h. A concurrent positive control (350 μg/mL ENU or 2.5 μg/mL B[*a*]P) was included in each experiment. Following treatment, the substance formulations were removed and washed off using HBSS, and the cells were incubated for an additional 72 h in SFM. After the 72‐h sampling period, cells were lysed either overnight with 3 mL DNA lysis buffer (10 mM Tris pH 7.6, 10 mM EDTA, 150 mM NaCl, 1% SDS, and 1 mg/mL proteinase K) for mutant frequency assessment, or for 1 h on an orbital shaker (100 rpm) with 1.5 mL lysis buffer I (0.584 mg/mL NaCl, 1 mg/mL sodium citrate, 0.5 μL/mL IGEPAL, 0.7 U/mL RNase A, and 0.5 μM SYTOX green nucleic acid stain), followed by an extra 30 min on an orbital shaker (100 rpm) after the addition of 1.5 mL lysis buffer II (85.6 mg/mL sucrose, 15 mg/mL citric acid, and 0.5 μM SYTOX green nucleic acid stain) for flow cytometric assessment of induced cytotoxicity (see Section 2.2.1.4).

DNA was isolated from cell lysates using phenol–chloroform extraction as described before (Cox et al. 2019b; Göpfert et al. 2025). Briefly, DNA isolation involved a phenol‐chloroform (1:1 by volume) purification step, followed by a chloroform‐isoamyl alcohol (24:1 by volume) step, including the addition of sodium chloride (final concentration of 200 mM). The final purification step involved a last round of chloroform‐isoamyl alcohol (24:1 by volume) purification. Ethanol was used to carefully precipitate strings of DNA, which were subsequently collected using heat‐sealed glass Pasteur pipettes. The collected strings of DNA were washed with freshly prepared 70% ethanol, air‐dried, and dissolved in 50–100 μL TE^−4^ buffer (10 mM Tris pH 7.6, 0.1 mM EDTA).

The DNA isolated from treated primary MutaMouse hepatocytes was then used to assemble viable lambda phages carrying mutated or non‐mutated *lacZ* reporter genes using the Transpack Packaging Extract for Lambda Transgenic Shuttle Vector Recovery from Agilent Technologies Inc. As described previously (Chen et al. 2010; Cox et al. 2019b), mutants were selected using the positive selection method with Phenyl‐β‐D‐galactopyranoside (P‐Gal). Therefore, *E. coli* C Δ*lacZ*, ΔgalE, ΔrecA, Kanr, pAA119 host cells were infected with the assembled phages, enabling the transfer of reporter genes into the host cells. Following infection, mutant phenotypes were selected by simultaneously plating infected cells in the absence and presence of P‐Gal. Plated cells were incubated overnight at 37°C. The next morning, the plaques that emerged under selective and non‐selective conditions were scored manually, and the mutant frequency (MF) was calculated by dividing the number of mutants determined from selective plates by the overall titer determined from non‐selective plates. A minimum titer of 100,000 total plaque forming units (PFU) was evaluated per sample.

##### 2.2.1.4 Cytotoxicity Assessment

As proposed by Cox et al. (2019b), the relative increase in nuclear counts (RINC) was used as a measure for cytotoxicity. RINC was quantified by flow cytometry as described previously (Göpfert et al. 2025). Briefly, lysates were spiked with a fixed concentration of 6 μm fluorescently labeled polystyrene beads prior to flow cytometric measurement. Data were acquired using the FACSLyric flow cytometer from BD Biosciences, equipped with a 488 nm laser. SYTOX Green and bead fluorescence emission were captured in the FITC channel (530/30 bandpass filter). Nuclei were gated based on their SYTOX Green signal. Measurement was ended after a maximum time of 480 s or once 10,000 events were detected in the nuclei gate. The following equations were used to calculate the nuclei counts and RINC values:

$$\begin{array}{c} \text{Nuclei count}\\ =\frac{\left({\text{population}}_{2N}*2\right)+\left({\text{population}}_{4N}*4\right)+\left({\text{population}}_{8N}*8\right)}{{\text{population}}_{\text{beads}}} \end{array}$$

$$\text{RINC}=\frac{{\text{Nuclei count}}_{\text{treated sample}}}{{\text{Nuclei count}}_{\text{vehicle control sample}}}$$

#### 2.2.2 In Vitro Mammalian Cell Gene Mutation Test Using the Hprt Gene

The In Vitro Mammalian Cell Gene Mutation Test using the Hprt gene (HPRT assay) was performed according to OECD TG no. 476.

##### 2.2.2.1 Cell Line

Chinese Hamster Ovary (CHO) cells were purchased from ICN Laboratories GmbH (Meckenheim, Germany) and maintained in the laboratory as master and working cell banks. Prior to freezing, the cells were pretreated with HAT (hypoxanthine, aminopterin, thymidine) supplemented medium (2% (v/v)) to eliminate any spontaneous HPRT‐deficient mutants and subcultured twice. Before use in the HPRT assay, cells were checked for mycoplasma contamination, then thawed and subcultured once more. Unless otherwise specified, the cells were maintained in Ham's F12 medium enriched with stable glutamine and hypoxanthine, 10% (v/v) fetal calf serum (FCS), 1% (v/v) penicillin–streptomycin, and 1% (v/v) amphotericin B. Incubation conditions during the assay were as follows: 37°C in an atmosphere of 5.0% (v/v) CO_2_ and a relative humidity of ≥ 90%.

##### 2.2.2.2 Mutagenicity Assessment

Briefly, 15 × 10^6^ CHO cells were seeded into 300 cm^2^ cell culture flasks. The next day, these cells were treated with various concentrations of test substance formulated in the respective vehicle (Table 1), the vehicle itself, or the positive control (in the absence of S9 mix: 400 μg/mL EMS; in the presence of S9 mix: 1.25 μg/mL DMBA). Depending on the mutagenic mechanism of the test compound, the 4‐h treatment was performed either in the absence or presence of an exogenous metabolic activation system (2% (v/v) S9 mix in culture medium without FCS) (Table 1). The S9 mix was freshly prepared prior to use as described by Ames et al. (1975). Briefly, one part of S9‐fraction (ICCR Roßdorf, Germany) was added to nine parts of S9 supplement (5 mM glucose‐6‐phosphate, 8 mM MgCl_2_, 33 mM KCl and 4 mM NADP in phosphate buffer (15 mM, pH 7.4)). Following treatment, cells were washed using HBSS, trypsinized (0.25%) and subcultured by seeding 2 × 10^6^ cells per 175 cm^2^ cell culture flask (Passage I). After 3 days of growth, cells were subcultured as before (Passage II) and cultured for an additional 4 days. On day seven, cells were seeded for selection. Specifically, 2 × 10^6^ cells from each treatment culture were seeded in duplicates into 175 cm^2^ cell culture flasks containing selection medium supplemented with 10 μg/mL 6‐Thioguanine (6‐TG). Selection was ended after 7 days, and colonies grown under selective conditions were fixed in the cell culture flasks for 5 min using methanol, then stained for 10 min with 10% Giemsa in deionized water. Residual staining solution was washed off with deionized water, and stained colonies were counted manually.

##### 2.2.2.3 Cytotoxicity Assessment

When cells were subcultured after treatment, 200 cells from each treatment culture were seeded in duplicate into 6 cm petri dishes for evaluating the first cloning efficiency (ce1, survival rate). Cell survival was assessed 7 days after plating by fixing and staining colonies as described above (5 min methanol fixation, 10 min 10% Giemsa staining). Stained colonies were counted using the Sorcerer Image Analysis System (Instem plc, UK). Cloning efficiency was evaluated a second time on the same day, when cells were seeded for mutant selection. The procedure for determining the second cloning efficiency (ce2) was identical to that for ce1.

#### 2.2.3 Statistics

Statistical evaluation of the *vitro*TGRA data was carried out using the SAS procedure GENMOD. The number of mutant plaques was analyzed for each sample using general linearized models. The technical replicate was set as the statistical unit. It was assumed that the number of plaques is Poisson‐distributed. The log link function was used to ensure the number of total PFUs was used as an offset. Pearson's chi‐square was used to estimate underdispersion and overdispersion. Pairwise comparisons of each dose with the vehicle control group were performed using a one‐sided likelihood ratio test with the hypothesis of equal mean values, applying the Bonferroni‐Holm correction. A linear trend test was performed using the assumptions described above. All tests were carried out one‐sided at significance levels of 1% and 5%.

Statistical evaluation of the HPRT assay was carried out using the Excel functions RGP and HYPGEOM.VERT. A linear concentration‐response was evaluated by testing for a linear trend. The independent variable was the concentration, and the dependent variable was the corrected mutant frequency. The calculation was performed using the RGP function in Excel. Pairwise comparisons of each concentration group with the vehicle control group were performed using Fisher's exact test with Bonferroni–Holm correction. The calculations were performed using the HYPGEOM.VERT function in Excel. All tests were carried out one‐sided at significance levels of 1% and 5%.

#### 2.2.4 Benchmark Concentration (BMC) Analysis

To assess potential differences in the response behavior of the two in vitro gene mutation assays (HPRT, *vitroTGRA*) at low concentrations, benchmark concentration analysis was performed. Benchmark concentrations (BMC) and their 90% confidence intervals (CIs)—shown as the lower (BMCL) and upper (BMCU) control limits—were estimated using PROAST, version 70.1 (RIVM web‐tool (https://proastweb.rivm.nl/)) for substances that tested positive in both assays. Using this tool, a default set of models (Null, Full, Exponential and Hill model families, inverse Exponential, and Log‐Normal model family) is fitted to the data. As suggested for genotoxicity endpoints, a benchmark response of a 50% increase in mutant frequency compared to the vehicle control was chosen (White et al. 2020). The concentration‐response curves were estimated using 200 bootstrap runs for model averaging, and the default setting of a maximum difference of 2 in the Akaike Information Criterion (AIC) was maintained to exclude unsuitable models.

## 3 Results

### 3.1 Impact of Cytotoxicity on the *vitroTGRA*

**FIGURE 1:**
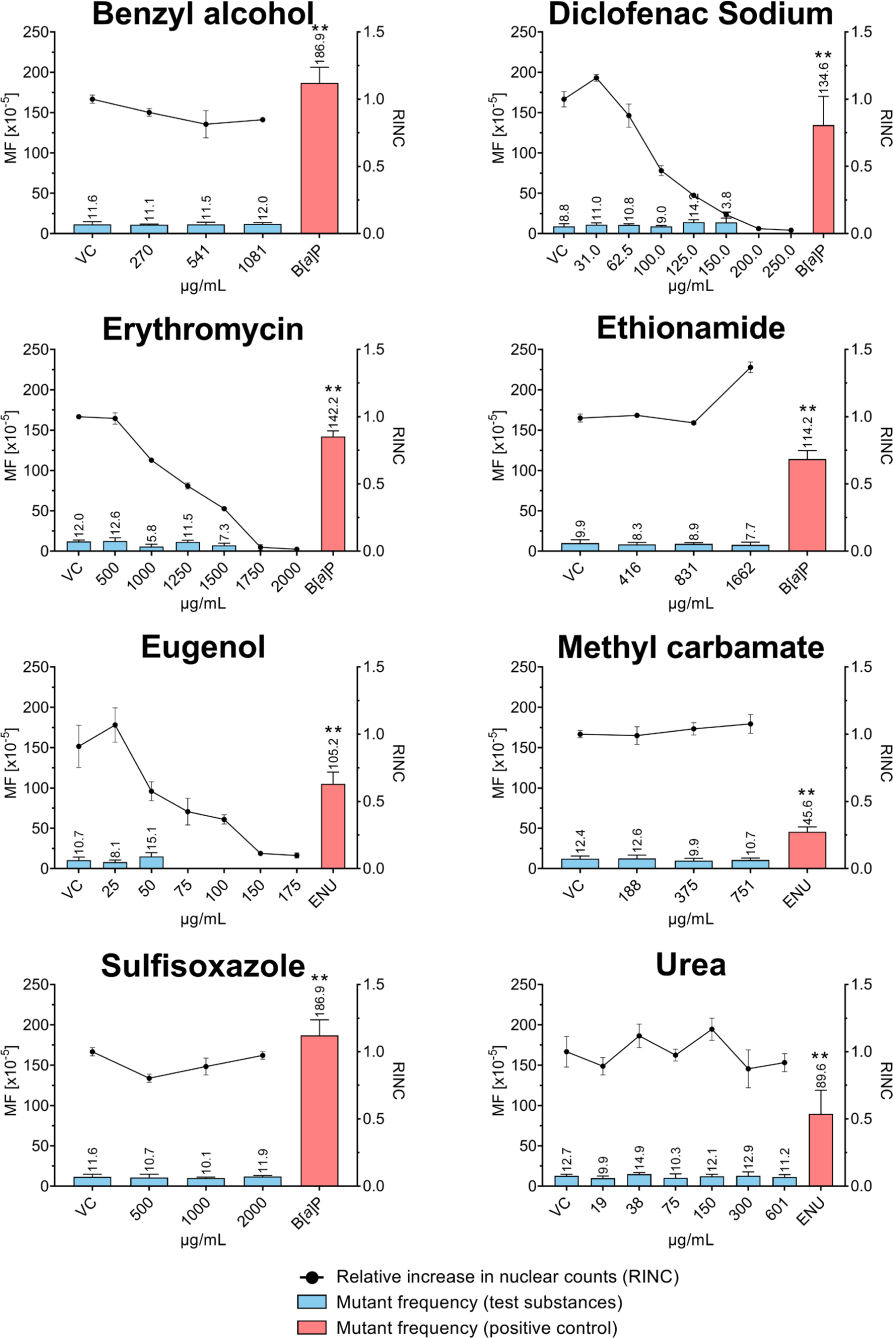
LacZ transgene mutant frequency (MF) in response to increasing concentrations of the eight in vivo non‐genotoxic substances tested (blue bars) and the concurrently run positive controls (red bars; either 2.5 μg/mL B[*a*]P or 350 μg/mL ENU). Induced cytotoxicity is presented as relative increase in nuclear counts (RINC, circles). The displayed values for MF and RINC represent the mean values with standard deviations obtained from three technical replicates in a single *vitroTGRA* experiment in primary MutaMouse hepatocytes. Asterisks indicate statistically significant increases in MF relative to the concurrent vehicle control (**p < 0.01; *p < 0.05).

This assessment was made to determine whether overt cytotoxicity alone affects the mutation frequencies in the *vitro*TGRA by using non‐DNA‐reactive substances. Benzyl alcohol, ethionamide, methyl carbamate and urea were tested up to 10 mM, while erythromycin and sulfisoxazole were tested up to a maximum concentration of 2 mg/mL. Of the eight substances, only diclofenac, erythromycin and eugenol induced clear cytotoxicity (Figure 1). Based on cytotoxicity observed in preliminary tests, the highest test concentration was set at 250 μg/mL for diclofenac and 175 μg/mL for eugenol. An amount of 150 μg/mL diclofenac (RINC 0.141 ± 0.012) was the highest concentration that was still suitable for mutant frequency assessment. At higher concentrations (RINC values ≤ 0.037), the DNA yield was insufficient to obtain a titer of 100,000 PFU. For erythromycin, the same observation was made at concentrations of 1750 μg/mL and higher (RINC values ≤ 0.029). For eugenol, a titer of 100,000 PFU was not reached at concentrations of 75 μg/mL and higher (RINC values ≤ 0.424).

None of the eight substances induced a significant increase in mutant frequency compared to the concurrent vehicle control (Figure 1). In addition, no statistical significance was observed in the trend test for any of the experiments. For vehicle control groups, the mean MF values in the eight experiments ranged from 9.9 × 10^−5^ to 12.7 × 10^−5^. For substance‐treated cultures, the mean MF values ranged from 5.8 × 10^−5^ to 15.1 × 10^−5^. All mean MF, after incubation with vehicle control or any of the test substances, were within the 95% limit of the laboratory's historical vehicle control data (Mean ± SD: 11.9 ± 4.3 × 10^−5^, 95% control limit: 2.9 × 10^−5^–21.0 × 10^−5^; *N* = 27). In all eight experiments, the concurrent positive control induced a significant increase in MF within the expected range.

### 3.2 Comparison of the *vitroTGRA* and HPRT Assay

**FIGURE 2:**
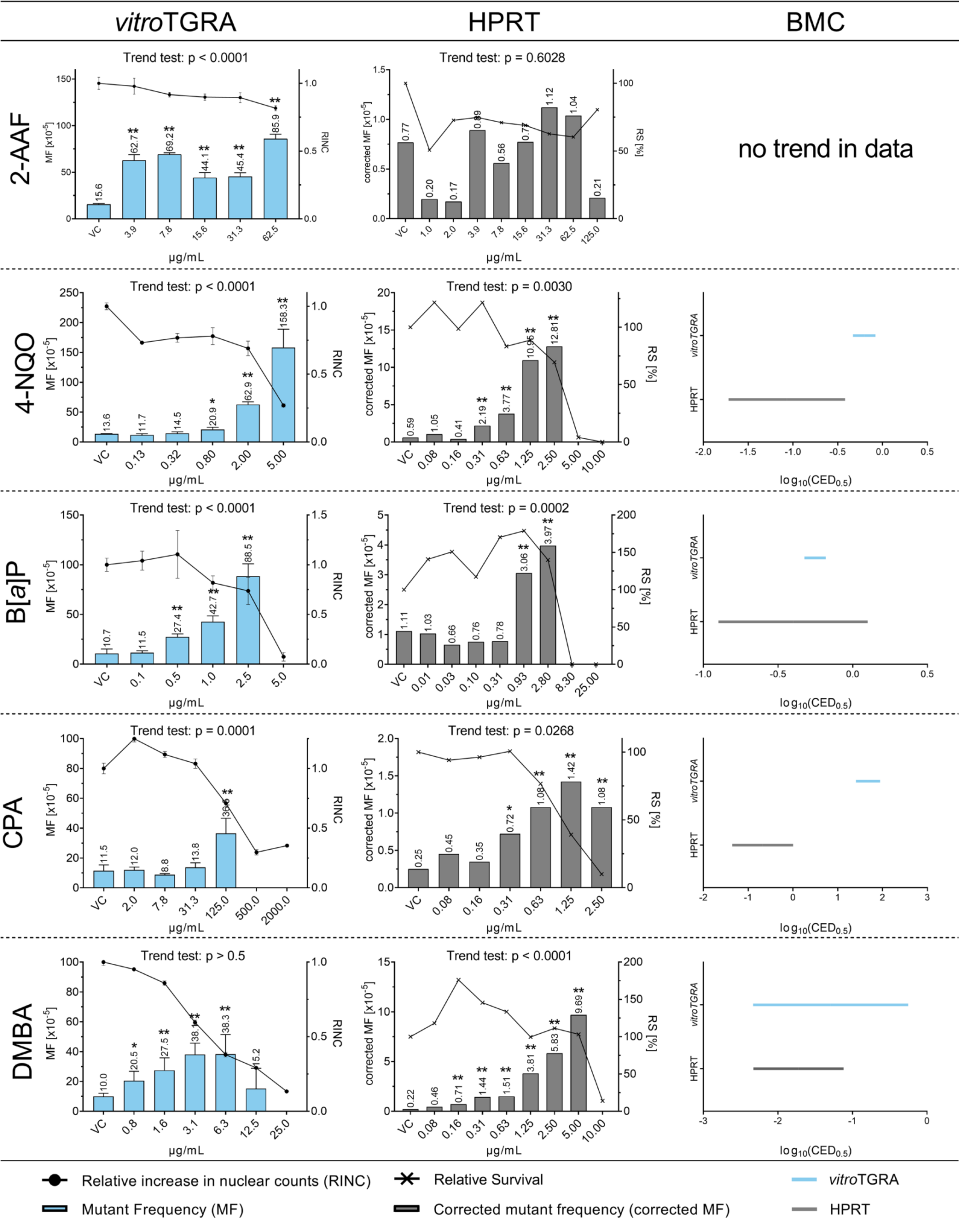
LacZ transgene mutant frequency (MF) (blue bars) and HPRT MF (gray bars) in response to increasing concentrations of the in vivo mutagens 2‐AAF, 4‐NQO, B[*a*]P, CPA and DMBA. Induced cytotoxicity is presented as relative increase in nuclear counts (RINC, circles) in the *vitro*TGRA and as relative survival (RS, crosses) in the HPRT assay. The displayed values for lacZ MF and RINC represent the mean values with standard deviations obtained from three technical replicates in a single *vitro*TGRA experiment. HPRT MF and RS represent values obtained from a single HPRT experiment. Asterisks indicate statistically significant increases in MF relative to the concurrent vehicle control (**p < 0.01; *p < 0.05). The log_10_ 90% BMC‐CIs of the estimated benchmark concentrations for a 50% increase in MF in the respective assay are presented as blue (*vitro*TGRA) and gray (HPRT assay) lines.

**FIGURE 3:**
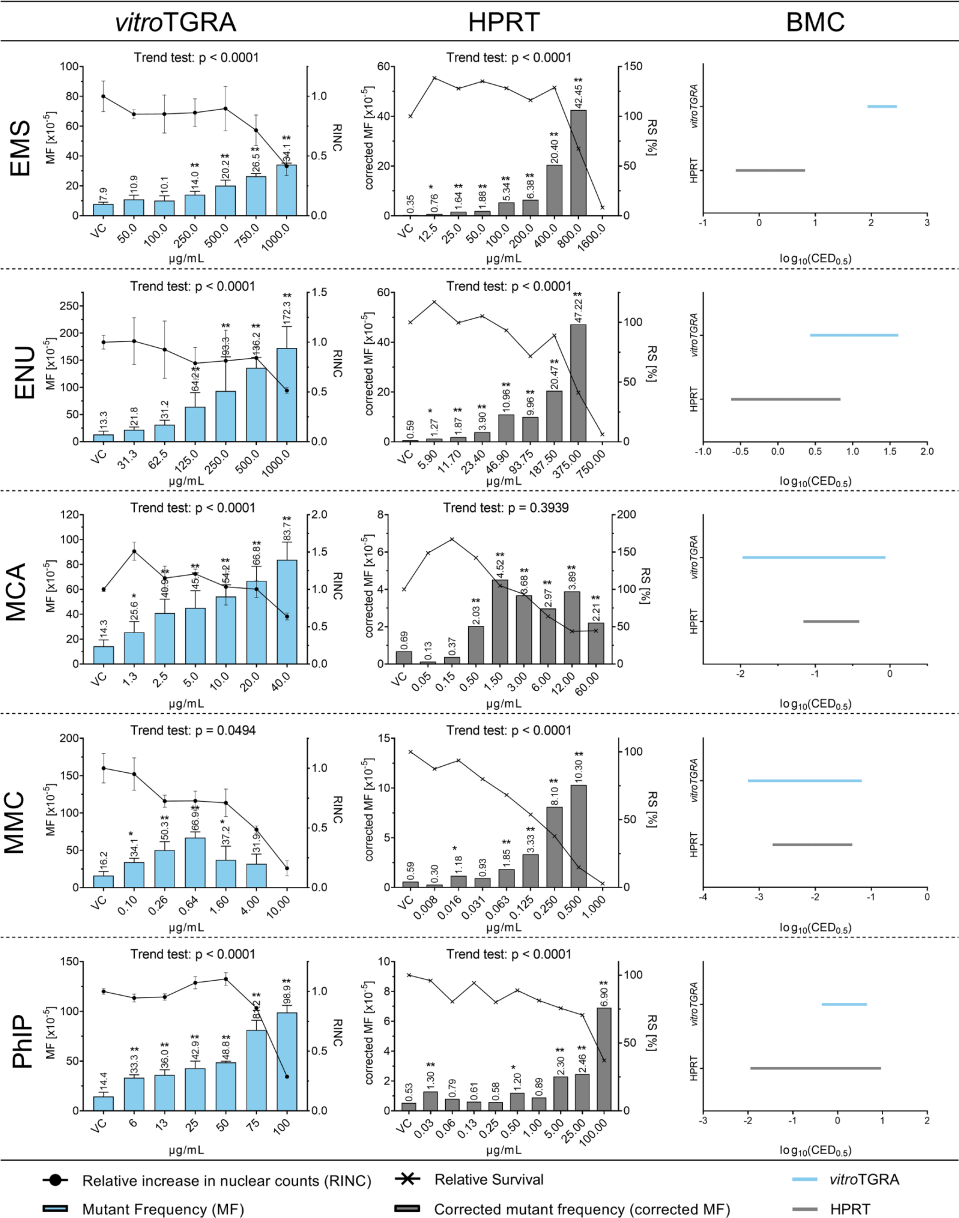
LacZ transgene mutant frequency (MF) (blue bars) and HPRT MF (gray bars) in response to increasing concentrations of the in vivo mutagens EMS, ENU, MCA, MMC, and PhIP. Induced cytotoxicity is presented as relative increase in nuclear counts (RINC, circles) in the *vitro*TGRA and as relative survival (RS, crosses) in the HPRT assay. The displayed values for lacZ MF and RINC represent the mean values with standard deviations obtained from three technical replicates in a single *vitro*TGRA experiment. HPRT MF and RS represent values obtained from a single HPRT experiment. Asterisks indicate statistically significant increases in MF relative to the concurrent vehicle control (**p < 0.01; *p < 0.05). The log_10_ 90% BMC‐CIs of the estimated benchmark concentrations for a 50% increase in MF in the respective assay are presented as blue (*vitro*TGRA) and gray (HPRT assay) lines.

To compare the *vitroTGRA* in primary MutaMouse hepatocytes and the HPRT assay in CHO cells, 10 in vivo genotoxicants (2‐AAF, 4‐NQO, B[*a*]P, CPA, DMBA, EMS, ENU, MCA, MMC, PhIP) were tested. For each test substance, a minimum of six test concentrations was applied. Cytotoxicity and (when possible) the mutagenic effect were determined. In addition, the BMC‐CIs (BMCL‐BMCU) were calculated for each test substance and assay and plotted in Figures 2 and 3.

In both *vitroTGRA* and HPRT assay, the spontaneous mutant frequencies of the vehicle controls were within the 95% limits of the laboratory's historical control data for each assay. Additionally, the positive controls induced statistically significant increases in mutant frequency (data not shown), confirming the validity of the experiments.

Of the 10 substances, all except 2‐AAF induced a statistically significant increase in MF at one or more test concentrations in both assays (Figures 2 and 3). In the *vitroTGRA*, 2‐AAF induced a statistically significant increase in MF at all tested concentrations (Figure 2) with MF values (44.1–85.9 × 10^−5^) exceeding the upper 95% limit of the laboratory's historical control data (Mean ± SD: 11.9 ± 4.3 × 10^−5^, 95% control limit: 2.9 × 10^−5^–21.0 × 10^−5^). In contrast, none of the 2‐AAF concentrations induced significant increases in MF in CHO cells in the HPRT assay. All MF values were within the 95% limit of the laboratory's historical control data range (with S9 mix: mean ± SD: 0.656 ± 0.275, 95% Limit: 0.059–1.252 × 10^−6^; without S9 mix: mean ± SD: 0.669 ± 0.329, 95% Limit: 0.000–1.435 × 10^−6^). Due to the negative result and the non‐significant trend in the HPRT data, BMC modeling was not performed for 2‐AAF.

4‐NQO induced a concentration‐dependent increase in MF in the *vitroTGRA*, reaching a maximum MF of 158.3 × 10^−5^. Cytotoxicity increased with higher concentrations. However, concentrations up to 5.0 μg 4‐NQO/mL yielded sufficient DNA to assess the mutagenicity. In contrast, 5.0 and 10.0 μg 4‐NQO/mL could not be evaluated in the HPRT assay, as these concentrations fell below the threshold of 20% relative survival. However, the MF was significantly increased (12.81 × 10^−5^) in the HPRT assay at 2.5 μg/mL. Since 4‐NQO tested positive in both assays, BMC analysis was performed. The BMC‐CIs estimated for the two assays did not overlap, indicating a difference in their response toward the mutagenic effect of 4‐NQO. According to the estimated BMC‐CI's, the HPRT assay was more sensitive to 4‐NQO induced mutagenicity than the *vitroTGRA*.

In both assays, B[*a*]P was tested up to similar concentrations (2.5 μg/mL in the *vitroTGRA* and 2.8 μg/mL in the HPRT assay). Both assays showed a concentration‐dependent increase in cytotoxicity and a statistically significant increase in mutant frequency, with a significant trend in the data. The estimated BMC‐CIs were overlapping, indicating comparable responses to B[*a*]P in the two in vitro gene mutation assays, whereby the confidence interval calculated for the HPRT assay was much broader than that for the *vitroTGRA*.

For CPA, the concentrations tested differed between the two assays. In the *vitroTGRA* concentrations of up to 125 μg CPA/mL were evaluable, while in the HPRT assay, the relative survival dropped to 10% at 2.5 μg CPA/mL. Concentrations from 0.31 to 2.5 μg CPA/mL induced statistically significant increases in MF in the HPRT assay, but all MF values (0.72–1.42 × 10^−5^) were within the laboratory's historical control range (0.251–1.569 × 10^−5^). In the *vitroTGRA*, the increase in MF at 125.0 μg CPA/mL was statistically significant and above the 95% limit of the laboratory's historical control data. Differences between the two assays were evident at low concentrations. The estimated BMC_50_‐CI's of both assays did not overlap, indicating that the HPRT assay was more sensitive in the detection of CPA‐induced mutagenicity than the *vitroTGRA*.

The evaluable concentrations of DMBA differed only slightly between the two assays: In the HPRT assay, 5.0 μg DMBA/mL was the highest evaluable test concentration based on the relative survival, while in the *vitroTGRA* DMBA could be tested up to 12.5 μg/mL. Both assays showed a statistically significant, concentration‐dependent increase in MF and the highest MF values exceeded the 95% limit of the laboratory's historical control data. The BMC_50_‐CIs of DMBA overlapped.

EMS induced a statistically significant, concentration‐dependent increase in the MF in both assays. The induced MF exceeded the 95% limit of the laboratory's historical control data for each assay. The estimated BMC_50_‐CI's did not overlap; the HPRT assay was more sensitive than the *vitro*TGRA.

In the *vitroTGRA*, ENU could be tested up to 1000 μg ENU/mL, whereas in the HPRT assay, 750 μg ENU/mL induced cytotoxicity with a relative survival of 7.9%. Nevertheless, statistically significant, concentration‐dependent increases in MF were observed in both assays, with values above the 95% limit of the laboratory's historical control data. The BMC_50_‐CI overlapped.

MMC was another substance that could be tested at higher concentrations in the *vitroTGRA* than in the HPRT assay. In the *vitroTGR*, 10 μg MMC/mL induced cytotoxicity that prevented sufficient DNA yield for MF assessment. In contrast, in the HPRT assay, 1.0 μg/mL MMC induced cytotoxicity that rendered MF assessment impossible. In both assays, a statistically significant increase in MF was observed, with values exceeding the 95% limit of the laboratory's historical control data. The BMC_50_‐CI's of both assays overlapped.

PhIP and MCA did not induce cytotoxicity in either of the two assays, which impeded MF assessment. Nevertheless, statistically significant, concentration‐dependent increases in MF above the 95% limit of the laboratory's historical control data were observed in both assays. The 90% CIs of the BMCs overlapped.

## 4 Discussion

As originally described by Cox et al. (2019a, 2019b), primary mouse hepatocytes are commonly isolated using retrograde liver perfusion. This technique was also previously employed by this laboratory (Göpfert et al. 2025). This sophisticated isolation procedure is technically demanding, requires well‐trained staff, and often results in highly variable hepatocyte yield and viability. These factors limit the feasibility of this technique in many laboratories. In 2022, Poggel et al. (2022) introduced a semi‐automated perfusion device for generating single‐cell suspensions from rodent livers. This gentleMACS Perfusion Technology was tested as an alternative to the two‐step retrograde liver perfusion method for obtaining MutaMouse PHs. This new technology gave reproducible yields of viable primary hepatocytes. Cell viabilities greater than 80% were achieved in nearly all experiments, whereas retrograde liver perfusions rarely yielded hepatocyte populations with viabilities of 80% or higher. When working with mouse liver, the manufacturer recommends the perfusion of the left lateral lobe, rather than perfusing the whole liver. However, this would reduce the number of harvested hepatocytes and thus increase the number of animals required per experiment. In this study, the left and right lateral lobes of each mouse were perfused.

Isolation of primary hepatocytes by retrograde perfusion requires cannulating the veins of anesthetized animals, an animal experiment that places a burden on the animals. Therefore, performing retrograde liver perfusion requires review and approval from the animal welfare committees in many countries (Poggel et al. 2022). With the GentleMACS perfusion technology, primary hepatocytes are isolated after organ removal. Thus, this new technique refines the isolation procedure by reducing the burden on the animals and, hence, supports the 3R principle of Russell and Burch.

In other in vitro gene mutation assays, such as the in vitro mammalian cell gene mutation assays using the Hprt, Xprt, and thymidine kinase genes, the OECD TGs recommend using 10 mM, 2 mg/mL or 2 μL/mL (whichever is the lowest) as the highest test concentration in cases when the test substance does not induce precipitation and/or limiting cytotoxicity (OECD 2016a, 2016b). When testing poorly soluble substances, turbidity or a precipitate should be present at the highest test concentration. For cytotoxic substances, the OECD TGs stipulate that the highest test concentration should induce a relative survival or relative total growth of 10%–20%. Cox et al. (2019b) proposed that, in accordance with these test guidelines, MF values from test groups with RINC values less than 0.2 should be interpreted with caution in the *vitroTGRA*. However, it has not yet been investigated whether this threshold is also appropriate for the *vitroTGRA*. For the eight non‐DNA reactive substances tested, it was shown that excessive cytotoxicity limits the DNA yield and thus prevents mutagenicity assessment. At all other cytotoxicity levels, regardless of magnitude, the MF was not influenced by cytotoxicity. All eight substances tested negative, even at test concentrations inducing cytotoxicity with RINC values < 0.2 (relative increase in nuclear counts < 20%). These results indicate that cytotoxicity does not lead to spurious increases in MF; thus a cytotoxicity threshold may not be necessary; however, excessive cytotoxicity can impede mutagenicity assessment due to limited DNA yield.

Ten test substances known to be mutagenic in vivo were tested in the *vitroTGRA* and the HPRT assay (OECD TG no. 476). Nine of the 10 showed mutagenic effects in both assays; however, 2‐AAF, showed mutagenic effects only in the *vitroTGRA*. In the in vivo TGRA, 2‐AAF induced mutations in the liver and bladder (OECD 2009). Thus, the results of the *vitroTGRA* are in line with the corresponding in vivo study. 2‐AAF did not show a mutagenic effect in the HPRT assay. According to Kirkland et al. (2016), variable responses for 2‐AAF in the HPRT assay have been reported previously, whereas it tested positive in the bacterial reverse mutation test and the mammalian cell gene mutation assay using the TK gene (OECD 2009).

Beyond a qualitative comparison of the results from the *vitro*TGRA and HPRT assays, mutagenic potencies were also compared by calculating the BMC_50_. For six of the nine mutagens that tested positive in both assays, the BMC confidence intervals (BMC‐CI) overlapped, indicating similar potency in the two in vitro gene mutation assays. However, for CPA, EMS and 4‐NQO, the BMC‐CIs did not overlap. In all three cases, the BMC‐CIs estimated for the HPRT assay were lower than those for the *vitroTGRA*. One explanation for the difference in the estimated BMC‐CIs of EMS and 4‐NQO could be the low spontaneous mutant frequency observed in the vehicle control in the respective HPRT experiments. The spontaneous mutant frequencies were close to the lower 95% limit of the laboratory's historical control data. A 50% increase in such low MF values results in MF values that are still within the natural fluctuations of spontaneously occurring mutations. Another explanation could be the endogenous xenobiotic metabolizing capacity of the primary hepatocytes used in the *vitro*TGRA, in contrast to the limited or lacking capacity of CHO cells used in the HPRT assay, which rely on exogenous S9. Endogenous metabolizing capacity is considered advantageous because metabolites are formed within the cell and are per se bioavailable. Furthermore, these cells bear the complete set of metabolizing enzymes (Phase 1 and 2), unlike the exogenous S9, which is reliant on the activating co‐factors added. Primary hepatocytes used in the *vitroTGRA* maintain their xenobiotic metabolizing competence up to 24 h post isolation (Cox et al. 2019a). Since treatment in the *vitro*TGRA is performed within this time frame, CPA, EMS, and 4‐NQO could be detoxified by the hepatocytes. This is supported by the observed lower cytotoxicity, which allows evaluation of higher test concentrations in the *vitroTGRA* compared to the HPRT assay. Additionally, Zwart et al. (2012) showed, that primary hepatocytes from the pUR288 LacZ Plasmid Mouse have a properly functioning p53 gene, a gene encoding for a transcription factor that promotes cell cycle arrest, DNA repair, or apoptosis to prevent the accumulation of genetic lesions (Ozaki and Nakagawara 2011; Zwart et al. 2012). In p53‐competent cells, this transcription factor is activated upon DNA damage (Ozaki and Nakagawara 2011). Therefore, not all genetic lesions induced in cells with a functional p53 gene will result in mutations. This could make primary MutaMouse hepatocytes more resilient to mutagenic effects compared to CHO cells used in the HPRT assay. The higher sensitivity observed when using CHO cells lacking xenobiotic‐metabolizing capacity and p53 functionality may also explain the high false positive rate of in vitro mammalian cell gene mutation assays, as observed by Fowler et al. (2024). In their study, 15 out of 18 plant production products tested positive in in vitro mammalian cell gene mutation assays but were negative in the follow‐up in vivo studies. In the bacterial gene mutation assay (Ames test), only 2 of the 18 plant protection products gave false positive results. Further comparative studies should test substances known to be non‐mutagenic in vivo but reported to give false positive results in other in vitro gene mutation assays.

Overall, the *vitro*TGRA identified all 10 in vivo mutagens, whereas the HPRT failed to identify one of them (2‐AAF). The mutagenic potency of three test substances was higher in the HPRT assay than in the *vitroTGRA*. This did not prevent the identification of their mutagenic potential in the *vitroTGRA* and may be due to the presence of a functional p53 gene and endogenous xenobiotic‐metabolizing capacity, which more closely resembles the actual in vivo situation.

These data further validate the *vitroTGRA* as an effective in vitro assay for detecting gene mutations and provide essential information for its prevalidation.

## Author Contributions

N.H. and R.L. designed the study. A.G. contributed to the study design and performed the experiments. K.K.M.R., C.R., and S.S. assisted in the performance of the experiments. A.G. prepared the manuscript draft, including draft figures and tables. N.H. and R.L. gave important intellectual input to the manuscript. All authors had complete access to the study data and reviewed and approved the final manuscript.

## Conflicts of Interest

The authors declare no conflicts of interest.

## Data Availability

The data that support the findings of this study are available from the corresponding author upon reasonable request.
